# The diagnostic value of pepsin detection in saliva for gastro-esophageal reflux disease: a preliminary study from China

**DOI:** 10.1186/s12876-017-0667-9

**Published:** 2017-10-17

**Authors:** Xing Du, Feng Wang, Zhiwei Hu, Jimin Wu, Zhonggao Wang, Chao Yan, Chao Zhang, Juan Tang

**Affiliations:** 1 Department of Vascular Surgery, Xuan Wu Hospital, Capital Medical University, Beijing, 100053 China; 2 Department of Gastroesophageal Reflux Disease, PLA Rocket Force General Hospital, Beijing, 100088 China; 3 Department of General Surgery, Xuan Wu Hospital, Capital Medical University, Beijing, 100053 China; 4 Acupuncture and Moxibustion School of Teaching Hospital of Chengdu University of TCM, Chengdu, Sichuan 610097 China

**Keywords:** Gastro-esophageal reflux disease, Pepsin, 24-h multichannel intraluminal impedance pH monitoring, Endoscopy, Diagnosis

## Abstract

**Background:**

None of current diagnostic methods has been proven to be a reliable tool for gastro-esophageal reflux disease (GERD). Pepsin in saliva has been proposed as a promising diagnostic biomarker for gastro-esophageal reflux. We aimed to determine the diagnostic value of salivary pepsin detection for GERD.

**Methods:**

Two hundred and fifty patients with symptoms suggestive of GERD and 35 asymptomatic healthy volunteers provided saliva on morning waking, after lunch and dinner for pepsin determination using the Peptest lateral flow device. All patients underwent 24-h multichannel intraluminal impedance pH (24-h MII-pH) monitoring and upper gastrointestinal endoscopy. Based on 24-h MII-pH and endoscopy study, patients were defined as GERD (abnormal MII-pH results and/or reflux esophagitis) and non-GERD otherwise.

**Results:**

Patients with GERD had a higher prevalence of pepsin in saliva and higher pepsin concentration than patients with non-GERD and healthy controls (*P* < 0.001 for all). The pepsin test had a sensitivity of 73% and a specificity of 88.3% for diagnosing GERD using the optimal cut-off value of 76 ng/mL. Postprandial saliva samples collected when the symptoms occurred had a more powerful ability to identify GERD.

**Conclusions:**

Salivary pepsin test had moderate diagnostic value for GERD. It may be a promising tool to replace the use of currently invasive tools with advantages of non-invasive, easy to perform and cost effective.

**Trial registration:**

ChiCTR-DDD-16009506 (date of registration: October 20, 2016).

## Background

Gastro-esophageal reflux disease (GERD) refers to “a condition that develops when the reflux of stomach contents causes troublesome symptoms and/or complications” in the Montreal Classification [1]. As one of the most common gastrointestinal diseases, its prevalence has increased in the last few decades [2, 3], leading to a considerable healthcare burden and low quality of life. Current methods employed in the diagnosis of GERD include GERD questionnaires, “PPI test”, endoscopy and ambulatory esophageal reflux monitoring. However, the sensitivity and specificity of such methods have been questioned. Recent studies showed that the PPI test and structured questionnaires did not obtain ideal sensitivity and specificity for diagnosis of GERD [4–6]. Endoscopy is not adequate since non-erosive reflux disease (NERD) is more prevalent than erosive reflux disease in the GERD population [7]. Reflux monitoring, including esophageal pH metry and impedance-pH metry, although currently used as the available gold standard, has a lower sensitivity in patients with NERD compared to those with reflux esophagitis and lacks reproducibility [8]. After all, none of the current approaches has been proven to be a reliable tool for GERD, and more specific, non-invasive and cost effective diagnostic methods are warranted.

Pepsin, a potential factor contributing to the mucosal tissues when gastro-esophageal reflux (GER) occurs, is a protease originating from pepsinogen synthesized by the gastric chief cells. Pepsin has been found in many different tissue samples such as laryngeal mucosa, paranasal sinus mucosa, saliva, middle ear effusion, tracheal secretions and bronchoalveolar lavage fluid [9–13]. The presence of pepsin in esophagus or more proximal sites is indicative of reflux, suggesting that pepsin may be used as a biomarker for the objective assessment of GERD. Some studies have shown that pepsin detection in the sputum and/or saliva can be regarded as a sensitive, non-invasive method for the diagnosis of the proximal reflux of gastric contents or laryngopharyngeal reflux (LPR) [14–17]. Thus, in the present study, we aimed to determine whether the use of pepsin determination in saliva could be useful for diagnosing GERD in the adult cohort from China with Peptest (RD Biomed Ltd., Hull, UK).

## Methods

### Subjects

Adult participants were consecutively enrolled in this prospective study at the Department of GERD, The General Hospital of the PLA Rocket Force, China. Patients who had at least eight weeks’ history of symptoms suggestive of GERD (for example heartburn, regurgitation, non-cardiac chest pain, chronic cough, asthma, throat irritation or clearing, globus sensation) were eligible for enrollment in this prospective study. Before enrollment in the present study, patients received consultancy from the departments of cardiology, pulmonology, or otorhinolaryngology according to their symptoms, and had been fully studied to rule out causes other than GERD. Exclusion criteria were: central system diseases, connective tissue diseases, psychiatric disorders, previous gastric or esophageal surgery, Zollinger-Ellison syndrome, esophageal stricture, achalasia, autoimmune diseases and collagen vascular diseases.

Asymptomatic healthy volunteers were recruited from Center of health Examination, the General Hospital of the PLA Rocket Force, China, as normal controls. Subjects were eligible if they were age ≥ 18 years without GERD symptoms. We excluded subjects with a history of previous gastric or esophageal surgery, a known esophageal motor disorder (e.g. achalasia, scleroderma), or psychiatric disorders. A detailed GerdQ questionnaire was completed by the investigations for each patient and asymptomatic healthy subject. Signed informed consent was obtained from all participants before the study and the study protocol was reviewed and approved by the institutional review boards of the General Hospital of the PLA Rocket Force (Beijing, China) and was registered in Chinese Clinical Trial Registry (Registration number: ChiCTR-DDD-16009506).

During the study, all patients received 24-h multichannel intraluminal impedance pH (24-h MII-pH) monitoring, upper gastrointestinal endoscopy, esophageal high-resolution manometry (HRM) and upper gastrointestinal imaging. Reflux esophagitis was graded based on Los Angeles classification and esophageal motility parameters (including resting lower esophageal sphincter (LES) pressure, resting upper esophageal sphincter (UES) pressure and hiatus hernia (HH)) in HRM were defined according to Chicago classification.

### 24-h MII-pH monitoring

Patients were instructed to discontinue their PPIs medications 7 days prior to reflux monitoring, and H_2_ receptor antagonists, prokinetic medications, and antacids 3 days prior to the study. After an overnight fast, an experienced staff positioned the MII-pH catheter (Sandhill Scientific, Highlands Ranch, CO, USA) incorporating a pH sensor and six impedance channels located 5 cm above the proximal border of the LES, identified using HRM. The six impedance sensors were positioned 3, 5, 7, 9, 15, and 17 cm above the sphincter. During data acquisition, patients were asked to record mealtimes and activities, and log their symptom events electronically. Data was analyzed with dedicated software (Bioview Analysis; Sandhill Scientific, Highlands Ranch, CO, USA). Each graphical tracting of all events was further scrutinized manually to ensure accurate reflux detection. A pH study was considered abnormal if DeMeester scores were ≥14.7, or acid exposure time (AET) ≥4.2%. A impedance portion was defined as abnormal if percent bolus exposure time (BET) was ≥1.4%, or number of all reflux episodes ≥73 [18–20].

According to the endoscopy and the MII-pH study, patients were defined as GERD if they had reflux esophagitis, or abnormal pH results, or abnormal impedance results, and as non-GERD otherwise.

### Salivary pepsin

Salivary sample collection: Subjects were given 30-mL sterile plastic tubes containing 0.5 ml 0.01 mol/L citric acid, pH 2.5 to collect saliva. Subjects were instructed to cough a few times to clear the saliva from the back of their throat and then spit it into the tubes. Subjects collected saliva on morning waking, 1–2 h after lunch and dinner. Before collecting the early morning sample, subjects were required to refrain from brushing their teeth, drinking or eating. Samples were transferred to the refrigerator at 4 °C and analyzed within 7 days.

Pepsin measurement: Saliva specimens were analyzed using the Peptest lateral flow device (LFD) (RD Biomed Ltd., Hull, UK). Plastic tubes were centrifuged for 5 min at 4000 rpm in a bench top centrifuge and 80 μL supernatants were draw up into an automated pipette. The 80 μL sample was then mixed with 240 μL migration butter solution for 10 s. And the 80 μL of the mixture was added to the well of the LFD containing 2 unique human monoclonal antibodies that detected and captured pepsin protein (specific to pepsin-3), with a lower limit of detection of 16 ng/mL and an upper limit of 500 ng/mL. The value of 16 ng/mL was used as a cut-off to consider a saliva sample positive for pepsin. All samples with pepsin concentration below this threshold were regarded to have 0 ng/mL and those above 500 ng/mL had 500 ng/mL in the results.

### Statistical analysis

The SPSS 19.0 statistical software package (IBM, Armonk, NY) and Prism V.5.0, GraphPad were used for data processing. Continuous data were summarized as Mean ± SD if normally distributed and as median (interquartile rang, IQR) otherwise, and categorical variables were summarized as counts and frequencies. The Kruskall-Wallis test was used to conduct multiple group comparisons for non-normal distributed data and chi-squared test was used for categorical parameter. Correlations between pepsin concentration and reflux variables were assessed using Spearman’s rank correlation as appropriate. Receiver operating characteristic (ROC) curve analysis was performed to determine an optimal cut-off value of salivary pepsin concentration and compare the predictive values of different pepsin cut-off concentrations to diagnose GERD. The optimal cut-off was chosen using Youden index. All tests of significance were 2-sides, with *P* < 0.05 considered statistically significant.

## Results

### Patient characteristic

Three hundred and twelve participants were recruited. Eighteen of the patients could not tolerate the endoscopy or reflux monitoring and 9 of the healthy controls chose to drop out of the protocol, and were excluded from the analysis. Finally, one hundred and twenty two symptomatic patients (53 male and 69 female; median age, 53 (44–60) years), 128 patients (58 male and 70 female; median age, 50 (42–63) years) and 35 asymptomatic subjects (18 male and 17 female; median age, 48 (42–69) years) were included in the GERD, non- GERD and healthy controls. Differences in the ages, body mass index and sex distribution of subjects among the three groups were not significant (*P* > 0.05 for all) (Table 1). Out of 250 patients, 84% patients reported heartburn and/or regurgitation, 7% patients chest pain, 5% patients throat clearing, 4% patients chronic cough or asthma as primary complains. There were significant differences between non-GERD and GERD patients regarding to the parameters of reflux monitoring and HMR and the presence of HH, except for resting UES pressure (Table 2). Among the GERD patients, there were 31/122(25.4%)subjects with NERD, 91/122 (74.6%) subjects with reflux esophagitis (LA-A = 26, LA-B = 36, LA-C = 17, LA-D = 12), 102/122 (83.6%) with abnormal pH testing and 72/122 (59.0%) with abnormal impedance results, and 35/122 (28.7%) with abnormal pH testing and impedance results.

**Table 1:** Baseline characteristics for subjects in controls and two groups of patients

Clinical Variables	Controls (*n* = 35)	Non-GERD (*n* = 128)	GERD (*n* = 122)	*P*
Gender (M/F)	18/17	58/70	53/69	0.7
Age (yr), median (IQR)	48 (42–69)	50 (42–63)	53 (44–60)	0.664
Age (yr), rang	18–85	19–81	24–79	–
BMI (kg/m^2^), mean±SD	27.0±3.9	27.7±4.2	28.7±4.0	0.41
GerdQ score, mean±SD	4.1±1.9	6.3±2.6	11.0±2.8	<0.001

*GERD* gastro-esophageal reflux disease, *M/F* male/female, *BMI* body mass index, *SD* standard deviation

**Table 2:** The results of 24-h MII-pH monitoring and esophageal HRM in 2 cohorts

Parameters	Non-GERD (*n* = 128)	GERD (*n* = 122)	*P*
24-h MII-pH monitoring			
Demeester score, median (IQR)	11.2 (8.8–13)	26.3 (14.0–51.7)	<0.001
AET (%), median (IQR)	3.1 (2.1–3.7)	8.9 (4–12.1)	<0.001
BET (%), median (IQR)	1.05 (0.8–1.2)	2 (0.4–5.5)	0.006
Number of all reflux episodes, median (IQR)	63 (51.3–69)	75 (55.8–117.3)	<0.001
HRM			
Resting LES pressure (mmHg), median ( IQR)	12.4 (7.3–18)	8.4 (4.4–12)	<0.001
Resting UES pressure (mmHg), median (IQR)	57.2 (34.2–84.9)	58.4 (39.6–80.7)	0.605
HH^#^ (%)	24.2	73.0	<0.001

*24-h MII-pH* 24-h multichannel intraluminal impedance pH, *IQR* interquartile rang, *GERD* gastro-esophageal reflux disease, *AET* acid exposure time, *BET* bolus exposure time, *HRM* high resolution manometry, *LES* lower esophageal sphincter, *UES* upper esophageal sphincter, *HH* hiatal hernia

^#^HH was detected by endoscopy combined with HRM

### Salivary pepsin results

**Fig. 1:**
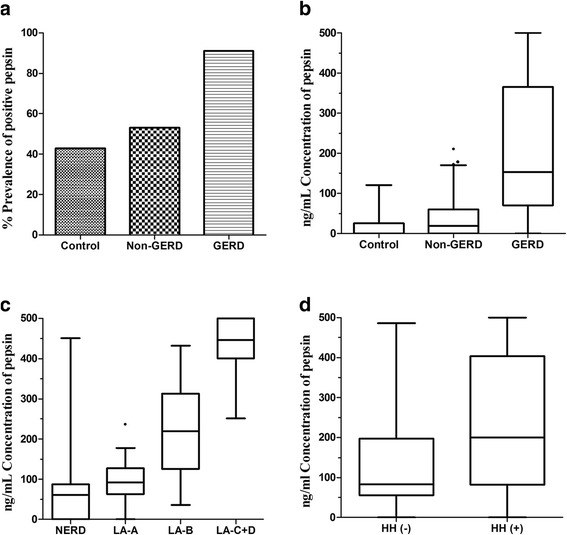
**a** The prevalence of positive pepsin in controls and two groups of patients. **b** The concentrations of pepsin in controls and two groups of patients. **c** The concentrations of pepsin in GERD patients with NERD, LA-A, LA-B and LA-C + D based on Los Angeles classification. **d** The concentrations of pepsin in GERD patients with or without HH. GERD: gastro-esophageal reflux disease, NERD: non-erosive reflux disease, HH: hiatus hernia

On the basis of the thresholds of 16 ng/mL, there was a significantly stepwise increase in the prevalence of positive pepsin among the 3 cohorts: results were positive in 111/122 (91.0%) of GRED who had at least one saliva sample positive for pepsin, 68/128 (53.1%) of non-GERD, and 15/35 (42.9%) of controls (*P* < 0.001) (Fig. 1a). The number of subjects having all three samples positive for pepsin was small (16/122 in GERD, 14/128 in non-GERD, and 4/35 in controls) (Table 3).

**Table 3:** The prevalence and concentrations of pepsin in saliva for controls and two groups of patients

Parameters	Controls (*n* = 35)	Non-GERD (*n* = 128)	GERD (*n* = 122)	*P*
Prevalence of positive samples (%)	29.5	32.3	62.0	<0.001
Proportion of subjects having at least one positive samples (%)	42.9	53.1	91.0	<0.001
Proportion of subjects having all three positive samples (%)	11.4	10.9	13.1	0.864
Concentrations of salivary pepsin (ng/mL), median (IQR)	0 (0–25)	18.5 (0–59.75)	153.3 (70–365.8)	<0.001

*GERD* gastro-esophageal reflux disease, *IQR* interquartile rang

The salivary pepsin concentration of each subject was determined as the highest value of all samples. The pepsin concentration was significantly highest in the GERD group (153.3 (70–365.8)) ng/mL, followed by non-GERD (18.5 (0–59.75) ng/mL), and lowest in controls (0 (0–25) ng/mL) (*P* < 0.001) (Table 3, Fig. 1b).

Overall, compared to postprandial samples, the positivity rates of pepsin and concentration of pepsin were significantly lower in the morning waking samples, both in non-GERD and GERD cohorts, while such differences were not found in controls (Table 4). Among patients including subjects with GERD and non-GERD, there were no statistically significant differences between samples after lunch and those after dinner, in terms of the prevalence of positive pepsin samples and concentration of salivary pepsin (Table 4). Interestingly, there were 27 saliva samples collected when symptoms occurred after meals from 27 GERD patients, which had higher positive rates of salivary pepsin and median concentrations of pepsin though statistically significant differences were not obtained compared to the overall postprandial samples (prevalence, 85.2% vs. 74.2%, *P* = 0.208; concentrations, 130 (36–392) ng/mL vs. 96 (0–278) ng/mL, *P* = 0.134).

**Table 4:** The prevalence and concentrations of pepsin in saliva at different sampling time points for three cohorts

Parameters	Morning waking	After lunch	After dinner	*P*
Control (*n* = 35)				
Prevalence (%)	17.1	37.1	34.3	0.14
Concentrations (ng/mL)^#^	0 (0–0)	0 (0–21)	0 (0–25)	0.095
Non-GERD (*n* = 128)				
Prevalence (%)	22.7	36.7	37.5	0.017
Concentrations (ng/mL) ^#^	0 (0–0)	0 (0–31.5)	0 (0–39.5)	0.005
GERD (*n* = 122)				
Prevalence (%)	37.7	73.8	74.6	<0.001
Concentrations (ng/mL) ^#^	0 (0–75.3)	103.5 (0–274.8)	92 (0–288.3)	<0.001

*GERD* gastro-esophageal reflux disease

^#^presented as median (IQR)

### The features of different values of pepsin concentration in saliva to differentiate patients with GERD from patients with non-GERD

**Fig. 2:**
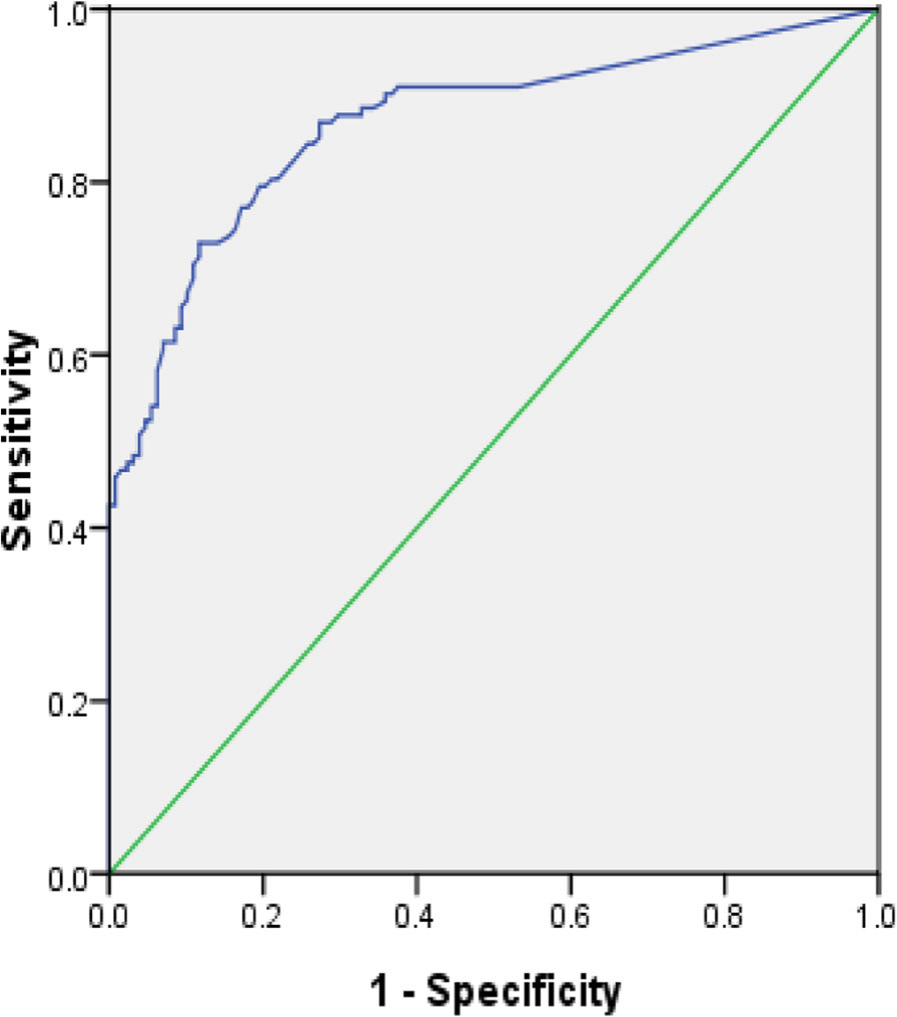
Receiver operating characteristic curve analysis for determining an optimal cut-off value of salivary pepsin concentration to identify patients with GERD. GERD: gastro-esophageal reflux disease

**Fig. 3:**
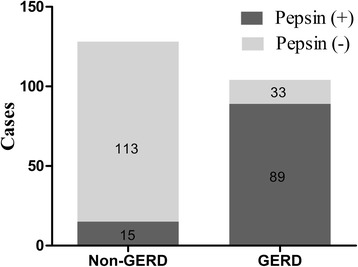
Comparison of salivary pepsin results with 24-h MII-pH monitoring combined with endoscopy. GERD: gastro-esophageal reflux disease, 24-h MII-pH: 24-h multichannel intraluminal impedance pH

Using the ROC curve, we identified the optimal cut-off value of salivary pepsin concentration to differentiate GERD patients from non-GERD patients (Fig. 2). The area under the ROC curve was 0.868±0.023 (95% CI, 0.822 to 0.914, *P* < 0.001). When the best pepsin test cut-off value was determined to be 76 ng/mL, the value of Youden index was biggest (61.3%). And the sensitivity of the Peptest test was 73%, and the specificity was 88.3% at the measured optimal cut-off value (Fig. 3). In Table 5, we displayed a range of salivary pepsin concentrations and compared their predictive values to diagnose GERD using the endoscopy and MII-pH metry as the gold standard.

**Table 5:** The predictive values of a range of salivary pepsin concentrations to identify patients with GERD

Concentrations (ng/mL)	Sen (%)	Spe (%)	Youden index (%)	PPV (%)	NPV (%)	PLR	NLR
≥16	85.1	47.1	32.2	60.5	76.8	1.61	0.32
≥76	73.0	88.3	61.3	85.6	77.4	6.24	0.30
≥125	54.8	90.0	44.8	83.9	67.6	5.48	0.50
≥200	44.3	97.2	41.5	93.8	64.7	15.82	0.57

*GERD* gastro-esophageal reflux disease, *Sen* sensitivity, *Spe* specificity, *PPV* positive predictive value, *NPV* negative predictive value, *PLR* positive likelihood ratio, *NLR* negative likelihood ratio

### Correlation analyses between concentration of salivary pepsin, 24-h MII-pH monitoring and HRM

Spearman’s rank correlation analyses were used to evaluate potential correlations between concentration of salivary pepsin, reflux parameters and HRM. A complete list of the associations tested was presented in Table 6. These cases included the moderately positive correlation between the pepsin level and DeMeester score (r_s_ = 0.665, *P* < 0.001), the low positive correlation between the pepsin level and AET (r_s_ = 0.423, *P* < 0.001), and number of all reflux episodes (r_s_ = 0.424, *P* < 0.001), the very low positive correlation between the pepsin level and BET (r_s_ = 0.178, *P* = 0.005), the low negative correlation between the pepsin level and LES (r_s_ = -0.181, *P* = 0.004). There was no significant correlation between the pepsin level and UES (*P* = 0.376).

**Table 6:** Correlation analyses between concentrations of salivary pepsin, 24-h MII pH monitoring and HRM

Parameters	r_s_	*P*
Concentrations of pepsin		
vs. DeMeester score	0.665	<0.001
vs. AET	0.423	<0.001
vs. BET	0.178	0.005
vs. Number of all reflux episodes	0.424	<0.001
vs. LES	-0.181	0.004
vs. UES	−0.056	0.376

*HRM* high resolution manometry, *AET* acid exposure time, *BET* bolus exposure time, *LES* lower esophageal sphincter, *UES* upper esophageal sphincter, *24-h MII-pH* 24-h multichannel impedance pH

### Salivary pepsin in GERD

Among patients with GERD, there was also a stepwise increase in the concentration of salivary pepsin: highest in those with LA-C + D, followed by those with LA-B, lowest in those with LA-A and NERD (*P* < 0.001) (Fig. 1c). Similarly, subjects with HH had a high level of pepsin in saliva than those without HH (*P* = 0.005) (Fig. 1d).

## Discussion

GERD is a common but often misdiagnosed disease in clinical practice. Studies [21–28] showed that GERD was the underlying factor of many pulmonary and otolaryngology diseases and conditions, such as asthma, chronic cough, pneumonia, laryngitis, pharyngitis, hoarseness, and even malignant tumor of head and neck. Because of the nonspecific symptoms of reflux disease, the definitive diagnosis of GERD is still challenging. Currently, the most reliable test for GERD diagnosis is ambulatory 24-h MII-pH monitoring. But it has inherent limitations. First, a considerable proportion of GERD patients could not be detected via the MII-pH metry [29], and dietary modifications and activity restrictions during reflux monitoring may lead to false negative results. Second, there are some asymptomatic cases of reflux who will escape reflux-related monitoring. Third, it is an invasive, expensive technique and a proportion of patients could not tolerate this test. Therefore, the development of a noninvasive, convenient, effective and sensitive method is warranted in a clinical setting.

Pepsin has been proposed as a promising biomarker for GER [24, 30–32]. Several studies [33–37] have indicated that pepsin is a major cause of GER, particularly in weakly acidic and non-acidic reflux. Pepsin may be present in the saliva/sputum of patients during episodes of GER and can keep stable below pH 8.0 [38]. Its presence in the oropharynx suggests GER.

The Peptest test is a convenient, office-based, noninvasive and quick technique for pepsin determination with the use of two unique human monoclonal antibodies to capture pepsin-3b independent of pH, which is superior to the digestion assay [39]. Several studies have shown that the measurement of pepsin in saliva/sputum may be used as a promising tool for diagnosing LPR using the Peptest test [40, 41]. This article is the largest-scale, prospective study in which we assessed the diagnostic value of the Peptest test for objective GERD confirmed by 24-h MII pH monitoring combined with endoscopy. Our study showed that (1) patients with GERD had a higher prevalence of pepsin in saliva and higher pepsin concentration than patients with non-GERD and healthy controls, (2) Postmeal saliva samples collected at the time of symptomatic episodes had a higher positive rate for pepsin and level of pepsin than overall postprandial samples in GERD patients, (3) About 40% of healthy asymptomatic subjects had salivary pepsin with a low concentration of less than 130 ng/mL, (4) Using the cut-off value of 76 ng/mL, the sensitivity and specificity of the Peptest test were determined to be 73.0% and 88.3% respectively.

Kim et al. [14] used the western blot analysis to detect the pepsin in the sputum/saliva in patients with clinically suspected atypical GERD symptoms. The results found that the sensitivity and negative predictive value of pepsin determination were excellent in most of atypical symptom groups (100%), whereas its specificity and positive predictive value were relatively low (76%). Similar researches are few, so the significance of pepsin detection for adult patients with atypical symptoms should be further studied.

To date, three studies have assessed the value of salivary pepsin for GERD with heartburn in adult cohorts using the Peptest test [42–44]. In these three studies, MII-pH metry [43, 44] and pH metry combined with endoscopy [42] were utilized as diagnostic criteria for GERD or reflux-related symptoms. AET with or without reflux number was the only parameter that was used to determine whether reflux monitoring was abnormal, which would result in false negative results. By contrast, we used pH monitoring data (Demeester scores and AET), impedance study (BET and number of all reflux) and endoscopy as the basis for test characteristics, which had a higher sensitivity for detecting GERD.

In the study by Saritas Yuksel [42], salivary pepsin was positive in 12% of controls and 50% of objective GERD (abnormal AET and/or esophagitis). Pepsin assay had a low sensitivity of 50% and a high specificity of 92% using the cut-off of 50 ng/mL with only one saliva sample collected at a random time point. In the published study by Bortoli [43], the Peptest test was positive in 94% of NERD, similar to the results obtained by us, and the pepsin analysis was found to have a sensitivity of 100% and a specificity of 80%, superior to our results. So the authors proposed that salivary pepsin determination was a convenient, economic, accurate and highly specific test to detect GERD without esophagitis.

More recently, Hayat et al. [44] took three salivary samples for each participant on waking, 1 h after lunch and dinner during reflux monitoring period. They found that pepsin could be found saliva in healthy subjects and patients, and the postprandial saliva samples were more likely to be positive for pepsin and have a higher level of pepsin compared to the morning saliva samples. Both the prevalence and concentrations of salivary pepsin were significantly higher in patients with GERD and hypersensitive esophagus (HE) compared to those with functional heartburn (FH) and controls, with higher concentrations predicting a greater probability of reflux. These results were consistent with those obtained by us. However, there were some differences needed to be issued between the two studies. Compared to the study by Hayat et al., the prevalence and median concentrations of pepsin in saliva of GERD patients seemed to be higher in our study (prevalence, 78% vs. 91%; concentrations, 126 (49.7–246.3) ng/mL vs. 153.3 (70–365.7) ng/mL). Our optimal cut-off value of pepsin was lower than that obtained by Hayat et al. (207 ng/mL vs. 76 ng/mL), and the area under the ROC and the Youden index were bigger in the study by us. Namely, the results about the diagnostic value of the Peptest test in our study were superior to those obtained by Hayat. The differences might be explained by some reasons. First, we used MII-pH combined with endoscopy as gold standard to define GERD, which can low the false negative results. Such more “true” GERD patients could be detected with more significant differences on the pepsin concentrations between GERD patients and non-GERD patients. Second, postprandial saliva samples tend to have a higher positive rate of pepsin and level of pepsin compared to the morning waking samples by increased gastric pepsin concentration and volume of reflux after meals [24, 44]. In addition, Kim et al. [14] found that pepsin was significantly more likely to be detected at the time of symptoms than on the waking. In the present study, 27 samples taken at the symptoms after meals from GERD patients had a higher prevalence of pepsin and level of pepsin compared to the overall postprandial samples. Based on the evidences above, we think that postprandial salivary samples when the symptoms occur may have a more powerful ability of differentiating GERD patients from non-GERD patients and we recommend postprandial saliva sampling during the symptomatic phase in the clinical application. Third, the study protocol in our study might be different from that of Hayat.

Pepsin was found in saliva of 40% of healthy control subjects with a low concentration of pepsin as a result of physiological reflux. A higher concentration of pepsin and positive rate is more likely to capture GERD. In our study, all three negative saliva samples suggested a 76% probability of diagnosing non-GERD, whereas a positive samples ≥200 ng/mL suggested GERD with a probability of 94%.

Additionally, our study demonstrated that there were correlations between the level of pepsin and reflux parameters and esophageal motility (LES), suggesting that the pepsin test can be used as an indicator of degree of reflux. The Peptest analysis achieved the accepted sensitivity and specificity for diagnosing GERD. Though far from perfect, its intrigue is that it provides a non-invasive, easy to perform, and inexpensive technique different from the currently available tools.

There were some limitations in our study. First, a small number of healthy asymptomatic subjects were enrolled into this prospective study. Further large-scale population-based study is required to establish an appropriate normal value for healthy subjects. Second, our study did not measure reflux-symptom association analysis and did not classify non-GERD into HE and FH via this parameter because of the lack of definite consensus about the diagnostic criteria for HE and FH. Third, there was no follow-up data to observe pepsin change before and after antireflux therapy and we could not assess its predictive value for clinical outcome.

## Conclusions

In summary, as a rapid, convenient, cost effective and non-invasive method, the detection of salivary pepsin had moderate diagnostic value for GERD and may be a promising tool to replace the use of currently invasive tools.

## Abbreviations

24-h MII-pH: 24-h multichannel intraluminal impedance pH
AET: Acid exposure time
BET: Bolus exposure time
FH: Functional heartburn
GER: Gastro-esophageal reflux
GERD: Gastro-esophageal reflux disease
HE: Hypersensitive esophagus
HH: Hiatus hernia
HRM: High-resolution manometry
IQR: interquartile rang
LES: Lower esophageal sphincter
LFD: Lateral flow device
LPR: Laryngopharyngeal reflux
NERD: Non-erosive reflux disease
NLR: Negative likelihood ratio
NPV: Negative predictive value
PLR: positive likelihood ratio
PPV: positive predictive value
ROC: Receiver operating characteristic
SD: Standard deviation
Sen: Sensitivity
Spe: Specificity
UES: Upper esophageal sphincter
